# Physiology

**Published:** 1859-09

**Authors:** S. Weir Mitchell

**Affiliations:** Lecturer on Physiology in the Phila. Med. Association


### PHYSIOLOGY.

BY S. WEIR MITCHELL, M.D.,
LECTURER ON PHYSIOLOGY IN THE PHILA. MED. ASSOCIATION.

### I.—*Digestion of Albuminous Matters by the Pancreas.* By LUCIEN CORVISART.

Most of our readers will remember our recent notice of the paper of M. Corvisart. His essay has attracted much attention and some hostile criticisms. The following remarks by him upon his reviewers and experimental or other critics, explain some parts of his essay which were not altogether clear.

As regards certain experiments by Kefestein and Halwach, from which these gentlemen obtained results which they looked upon as adverse to the views of M. Corvisart, he speaks thus :—

The work presented by these gentlemen to the Academy of Sciences at Göttingen terminates with this conclusion: We entirely dissent from the views of M. Corvisart. The pancreatic juice is incapable of dissolving albumen. Now to the dictum of these gentlemen it would have been easy to oppose the results of my numerous experiments, which have been subjected to the strictest verification. Nevertheless, I determined upon offering to the Academy my attacked essay, and solicited them to accept for my reply an account of one experiment only, the details of which are as follows:—

The subject of the experiment was a young dog weighing about "twenty-four pounds," and previously deprived for the space of fifteen hours of both solid and liquid food: 34 grammes of hardened white of eggs, boiled in water for fifteen minutes, separated from the shells and yolk and roughly crumbled in a cloth, were put into the duodenum, and ligatures placed around its first and third portions. For the purpose of bringing about digestion simultaneously in the stomach, 20 grammes of the same albumen were placed in that viscus, all egress to the surface being prevented by the ligature at the first portion of the duodenum, and by another ligature placed around the clavical termination of the æsophagus. During the operation the pancreas was not disturbed in the slightest degree; indeed it was not even seen. Tubes were employed for the purpose of introducing the albumen at the same instant into the stomach and intestine, and all these operative precautions to which reference is made at page nine of my essay, and which seemed to me indispensable to the success of the operation, were most scrupulously observed. Fifteen hours afterwards, the dog was killed by strangulation. The duodenum presented a swollen, red, and turgid appearance; taken out of the abdominal cavity, and emptied of its contents, we found 150 grammes of a viscous liquid, of neutral, or at most of a feebly-alkaline reaction, and altogether destitute of putrefactive odor. The intestine displayed no traces of the coagulated albumen placed within it in the first instance, if we except five or six soft and attenuated fragments, recognizable, it is true, but not amounting in weight to so much even as 4 grammes.

(*a*) It follows, therefore, that the mixed fluid in the duodenum is capable of digesting albumen.

The stomach in the above experiment was found to contain 250 grammes of an acid liquid. The hard albumen had in like manner disappeared by solution.

The pancreas of the same dog, examined when the digestive process was at its acme, both in the stomach and in the duodenum, presented the following features: The color was of a faint pink. It showed no signs of tearing or of ecchymosis. It was removed, cut up into small pieces, and placed in 200 grammes of water, maintained for twenty-four hours in a closed glass vessel at a temperature varying from $7^{\circ}$ to $12^{\circ}$ Cent.; the product was then filtered, and I collected 180 grammes of a reddish, viscous fluid, which displayed neither a marked acidity, nor alkalinity to test-paper of extreme sensibility. This pancreatic infusion was tried upon ovalbumen, cooked as in the preceding experiment, and pounded. After remaining four hours only in the stove at a temperature of 40° Cent., the number of grammes of albumen which had disappeared amounting to 45 of the albumen employed in the first instance.

(*b*) Hence it follows that a simple infusion of pancreas can by a power of its own, and without any assistance from intestinal secretions, or bile, etc., digest a large quantity of coagulated albumen.

By the action of a few grammes of the infusion, I was enabled to assign to it a digestive power over fresh, uncooked fibrin, which, calculated proportionately for an infusion of the whole of a pancreas, would suffice for the digestion of 60 grammes of fibrin. Both the digestions, by means of pancreatic infusions and the vivisection itself, were performed in the presence of gentlemen at that time in Paris, viz. Dr. Kuhne, pupil of Messieurs Wohler and Wagner, and Dr. Snellen, of Utrecht, pupil of M. Donders. (Extract from my letter to the Acad. des Sciences de Göttingen; see Gött. Nachr., No. 6, March, 1859, and Zeitschrift für Rationellen Med. of Henlo and Pfeuffer, 1859.) It will be remarked that these several quantities—45 grains of albumen, 60 of fibrin—amount as nearly as possible to within a few grammes of the quantities I had specified in my essay two years before. Messrs. Kefestein and Halwachs have nevertheless affirmed that their experiments were more exact than any others that had been performed. But the exactness of their proceedings began only at that stage when the stove came into requisition; while in fact it was of the highest importance that such exactness should begin in the very abdomen of the animal whose function they sought to determine. These gentlemen were not sufficiently careful in guarding against fallacy. Firstly, inasmuch as they employed the pancreatic juice of an animal unfortunately laboring for eight days under a fistula; secondly, as they made infusions of the pancreas without being mindful of selecting the gland at a fixed and suitable period of the digestive process.

First. I had forewarned experimentalists in my paper that the results obtained by means of the tubes appended to the excretory canal—that is to say, the pancreatic fistulæ—would be so various that it would be impossible to arrive at any definite conclusions by means of them. Messrs. Kefestein and Halwachs continued to employ this mode of procedure in carrying out their first series of experiments. The result has been that they have secured none but negative results. They moreover placed themselves in conditions most unfavorable to success by an ill-judged excess of experimental zeal, by which they assigned the preference both in the collecting the juice and in the carrying out the experiment to that secretion of the pancreas poured out subsequently to an irritation lasting eight days without intermission, and conveyed by the tube, over that secretion formed immediately after the performance of the operation.

It is self-evident that the pancreatic juice collected almost at the moment of the operation alone approximates to the normal secretion, the first quantity which drains away being "that which was already secreted in its physiological integrity in the gland prior to the operation." The longer the interval permitted to elapse, the further does the secretion depart from the true physiological type. Every organ, in fact, possesses its own special sensibility. The eye cannot, like the mouth, tolerate the presence of a grain of sand. The pancreas in no way habituates itself to the existence of fistulæ, as does the stomach, designed, as is this latter, for the contact of foreign bodies.

On the one hand, pancreatic fistulæ, far from being capable of enduring for years, like those of the stomach, end fatally in a few days, or at most in a few weeks. On the other hand, in the case of a pancreatic fistula, the properties of the pancreatic juice begin to deteriorate considerably after a lapse of two, or at most of three days. This is owing either to a diminution in the weight of the solid constituents, or to an alteration in the properties of the secreted ferments without diminution of weight. By the eighth day the deterioration is at its maximum. At this time the pancreatic secretion is in the condition it assumes when it has been made to boil. It has lost all potency over albuminous bodies, although it is still capable of forming an emulsion with fat, and of imparting an alkaline reaction.

The mode of procedure adopted by Messrs. Kefestein and Halwachs with fistulæ will always be productive of negative results.

It is indispensable, when we desire to obtain pancreatic juice as nearly as possible in its normal condition, that that secretion be chosen which was formed in the gland prior to vivisection—that is to say, "that" juice which flows forthwith upon the operation.

"Upon the fulfillment of this condition depends the superiority of the infusion of a pancreas" taken from an animal at the moment of killing it; for if the pancreas be removed within a few seconds after the animal has been killed, the infusion takes hold of the juice normally secreted during life, and which has not yet escaped from the gland.

Secondly. This proceeding has furnished the material for the second series of experiments of Messrs. Kefestein and Halwachs.

But here again they have erred in a most marked degree. It is not sufficient that a secreting organ be taken in order to obtain its secretion forthwith upon the death of the animal. The gland must be secured at that moment when its secreting activity is at its height. This precaution has been neglected by Messrs. Kefestein and Halwachs; and to this omission is to be ascribed the confirmation of the negative results they had obtained. With regard to the experiments detailed in my former paper, I may state that they were performed with infusions of the pancreas taken from animals in whom the duodenum and stomach were full at the moment of killing. M. Meissner has distinctly affirmed that he has obtained active infusions by taking the precaution of securing the pancreas during the period of digestion—this precaution is indispensable. I may add, that if a young and healthy dog be fed with mixed and abundant diet, and that if it be killed toward the fifth or sixth hour after the meal, and the pancreas be then removed, the infusion of the gland will possess the highest* digestive activity.†

* At such a period of digestion the pancreatic juice is so energetic, that if one omits to arrest the infusion of this gland at the proper time, this latter, if it have been cut up in very small pieces, in part disappears, dissolved and digested by its own proper juice which has escaped from the channels in which normally it is confined during life.

† The infusion made in compliance with these conditions is frequently able to digest 20 or 30 grammes of fibrin in the cold, and in but a few hours, (10° Cent.)

There is in reality a fasting condition of the duodenum, which is not identical with the similar condition of the stomach; in like manner, the fasting condition of the stomach is not identical with that of the mouth. It is highly probable that some little fluid may escape from the pancreas immediately upon the reception of food from the stomach, but the period of greatest glandular activity, of the highest efficiency of the pancreatic juice, is at that moment when the stomach having performed its function, the duodenum begins to act in its turn. This period in the dog is attained toward the fifth or sixth hour. At that time, the stomach still contains some food, and the duodenum contains some already. Before this period is attained, the duodenum is still in its fasting condition, and the pancreas inactive; subsequently to it again, the pancreas becomes exhausted.

Montègre went so far as ostensibly to deny the digestive action of the gastric juice, and even its acidity, for the reason that he was accustomed to examine the secretion during the condition of fasting. The same error has induced Messrs. Kefestein and Halwachs to deny to the pancreatic juice all digestive power over albumen. Their good faith, it is to be observed, is entirely foreign to the question;* whoever imitates them will see, like them, negative results.

Thirdly. Researches of M. Meissner upon the function of the pancreas.

Following upon Messrs. Kefestein and Halwachs, Professor Meissner published in the "Zeitschrift für Rationellen Med." of April, 1859, (having previously read an account of them as early as the autumn of 1856, at the Scientific Congress of Carlsruhe,) the experiments which led him to affirm most positively not only the solution of albuminous bodies by the pancreas, apart from all putrefaction, but the transformation of these bodies into peptone, as I had previously maintained. M. Meissner states: The results at which I have arrived completely confirm those obtained by M. Corvisart, with this restriction, that it is necessary that the pancreatic juice be acid, and not neutral, alkaline, or acid indifferently.

I had in fact stated in the ninth proposition,—"The pancreatic juice possesses the special property of acting efficiently, whether in the alkaline, neutral or acid state."

I beg to refer to pages 8, 29, 32, and 33, of my essay, where is detailed the digestion of albumen brought about either naturally in the duodenum, or by employing the stove with pancreatic juice by itself, and most effectively performed, the reaction being neutral and even alkaline; and I would add, that I

---

* I would add, that on preparing for purposes of study an infusion of pancreas, it is necessary to avoid crushing the gland, or agitating it too frequently in the water, or protracting the infusion beyond the period when the liquor becomes clouded. Under all the circumstances, one perceives by this last sign that the juice is beginning to act upon the fatty matters in the gland itself; at a later period it will have begun to act upon the azotized substance, since, like the gastric juice, the pancreatic juice exhausts itself by agitation. Commonly, an infusion which is long in filtering and is cloudy has partly lost its efficiency, unless the operation is carried on at a very low temperature—seven to eight degrees Cent. Rapidity is the rule in the preparation of the infusion.

was led to assert the existence of this neutrality, not only from my belief in its verification, as far as albumen was concerned, but inasmuch as my experiments on digestion, repeated on fibrin, (pp. 36, 40, 42,) cellular tissue and gelatine, (pp. 67 and 78,) on muscular tissue and on caseine, (pp. 92, 98,) were attended by similar results. Moreover, in these comparative experiments I was not dealing with imponderable quantities, quantities difficult to estimate, but with 20, 30, or 40 grammes of the articles of food, the digestion of which was completed under the influence of an infusion of a dog's pancreas, alkaline, acid, or neutral. The object of M. Meissner led me to investigate anew whether the words "equally well" of my ninth proposition were rigorously correct. I have consulted the records of my experiments. I have taken into consideration the figures indicating the weight of albumen digested by some of the same pancreatic juice, (but varied in such a manner that one solution was neutral, a second alkaline, a third acid.) I noticed differences, it is true, but these amounted at most to a few grammes, and were so slight in themselves, that at this moment it would be impossible for me to say whether, 40 grammes of albumen being experimented upon, four grammes more are digested by the pancreatic secretion being acid or alkaline. This indifference was likewise displayed on placing food in the closed duodenum. At the moment the animal was killed, the reaction was found to be at one time acid, at another neutral, and a third time alkaline; the weight of the material digested under those varying conditions being subject to but little alteration. In conclusion, I would remark that during the experiment, the minutes of which have been drawn up, the attention of Messrs. Kuhne, Snellen, and myself was specially directed to the point in dispute. The minutes run as follows:—

With regard to the duodenum.—"The duodenum was found to contain 150 grammes of a viscous liquid, neutral or but very faintly alkaline, having no putrefactive smell, and showing no traces of the 34 grammes of the coagulated albumen placed within it in the first instance, with the exception of five or six soft fragments, which, though recognizable, did not amount in weight to as much as 4 grammes." With regard to the pancreatic infusion.—"After remaining four hours in the stove, the quantity of solid albumen which had disappeared amounting to 45 grammes of the albumen originally employed," and further, it is stated that "before the albumen was used, the infusion displayed to litmus or turmeric papers of great delicacy no noticeable traces either of acidity or alkalinity."

The weight attaching to the researches of M. Meissner urges me to solicit most earnestly for further investigations, which doubtless will not fail to be productive of some explanation of the cause of the discrepancy to which this special point has given rise between us.—(*Lancet*, No. 25, vol. i., 1859.)

II.—*On the Mode in which Sonorous Undulations are conducted from the Membrana Tympani to the Labyrinth in the Human Ear.* By JOSEPH TOYNBEE, F.R.S., Aural Surgeon to St. Mary's Hospital.

The opinion usually entertained by physiologists is, that two channels are requisite for the transmission of sonorous undulations to the labyrinth from the membrana tympani, viz.: the air in the tympanic cavity which transmits the undulations to the membrane of the fenestra rotunda and the cochlea, and, secondly, the chain of ossicles which conducts them to the vestibule. This opinion is, however, far from being universally received. Thus one writer contends that "the integrity of one fenestra may suffice for the exercise of hearing;"* another expresses his conviction "that the transmission of sound cannot take place through the ossicula;"† while Sir John Herschel, in speaking of the ossicles, says "they are so far from being essential to hearing, that when the tympanum is destroyed, and the chain in consquence hangs loose, deafness does not follow."‡ The object of this paper is to decide by experiments how far the ossicles are requisite for the performance of the function of hearing. The subject is considered under two heads, viz.: First, whether sonorous undulations from the external meatus can reach the labyrinth without the aid of the ossicles as a medium; second, whether any peculiarity in the conformation of the chain of ossicles precludes the passage of sonorous undulations through it.

1st. Can sonorus undulations reach the labyrinth from the external meatus without the aid of the ossicles as a medium? This question has often been answered in the affirmative, apparently because it has been ascertained that in cases where two bones of the chain have been removed by disease, the hearing power is but slightly diminished. In opposition to this view, it must, however, be remembered that the absence of the stapes is always followed by local deafness, while a fixed condition of this bone (anchylosis) is accompanied by very serious deafness. The following experiments, selected from several others, demonstrating the great facility with which sonorous undulations pass from the air to a solid body, indicate that the stapes, even when isolated from the other bones of the chain, may still be a medium for the transmission of sounds to the fenestra ovalis and the vestibule.

Experiment 1.—Both ears having been closed, a piece of wood, five inches long and half an inch in diameter, was held between the teeth, and a vibrating tuning-fork, $C^1$, having been brought within the eighth of an inch of its free extremity, its sound was distinctly heard and continued to be heard for between five and six seconds.

Experiment 2.—Three portions of wood, of the same length and thickness as that used in the previous experiment, were glued together so as to form a triangle, somewhat of the shape of the stapes; the base of this triangle being placed against the outer surface of the tragus, the tuning-fork, $C^1$, vibrating within a quarter of an inch from its apex, was heard for twelve seconds.

2d. Is there any peculiarity in the construction of the chain of ossicles to prevent the passage of sonorous undulations through them? This question has also been answered in the affirmative, on account of the various planes existing in this chain and of the joints between the several bones composing it.§ The following experiments, selected from a variety detailed in the paper, indicate

---

* Mr. Wharton Jones's Encyclopedia of Surgery, "Diseases of the Ear," p. 23.

† Mr. Brooke, Lancet, 1843, p. 380.

‡ Encyclopedia Metropolitana, article "Sound," p. 810.

§ Mr. Brooke, loc. cit.

that neither the variety of the planes existing in the chain, nor the presence of joints, prevents the passage of sonorous undulations through it.

Experiment 1.—Two pieces of wood, each five inches long, were glued together so as to represent the planes of the malleus and incus, a triangular piece similar to that used in the last experiment being glued to one surface of the interior extremity of the portion representing the incus so as to imitate the plane of the stapes. Three pieces of wood, each five inches long, were also glued together, end to end, so as to form a straight rod. The vibrating tuning-fork, $C^1$, being placed at one extremity of the apparatus representing the chain of bones, and the other end being placed between the teeth, the sound was heard most distinctly for several seconds; and when it ceased to be heard, the straight rod was substituted, and the sound was again heard, but only for three seconds.

Experiment 2.—Between each of the three pieces of wood representing the chain of ossicles, similar to those used in the previous experiments, were placed instead of glue two layers of India-rubber, about as thick as ordinary writing paper; the pieces of wood being held together, the tuning-fork placed at one end of the chain was heard as distinctly and as long as in the previous experiment. The experiments, dissections, and observations recorded in the paper induce the author to arrive at the following conclusions:—

1st. That the commonly received opinion, that sonorous undulations pass to the vestibule, through the chain of ossicles, is correct.

2d. That the stapes, even when disconnected from the incus, can still conduct sonorous undulations to the vestibule from the air. So far as our present experience extends, it appears that in the human ear sound cannot reach the labyrinth from the membrana tympani without the agency of two media, viz., the air in the tympanic cavity, and the chain of ossicles.—(*Lancet*, No. 25, vol. i., 1859.)

### III.—*On the Identity of the Meconium and Vernix Caseosa.* By PROFESSOR FORSTER.

The general opinion respecting the meconium is, that it consists in a mixture of bile, intestinal mucus, and intestinal epithelium; but microscopical examination shows that, besides the coloring matter of the bile, it is composed chiefly of the vernix caseosa. For the most part it consists of small, flat scales, which present all the characteristics of horny epithelial plates, completely corresponding to the horny scales of the vernix. Under the microscope, the meconium only differs from the vernix by the presence of the yellow coloring matter and the smaller number of fat globules. A proof of the identity, is its containing minute hairs, in just the same numbers as the vernix, which, indeed, without the microscope, may be separated from it by a needle. The horny scales could have no other source than the vernix, for the stomach and intestinal canal are lined with cylinder epithelium, and the mucous membrane of the mouth and œsophagus does not give rise to them. Besides these scales, we observe in the meconium fatty globules of different sizes, crystals of cholesterine, and irregular yellow and brownish clodlets, which give the dark color to the meconium, and are doubtless biliary coloring matter. The fatty globules are evidently of cutaneous sebaceous matter, and the cholesterine is in part derived from the bile, and in part from the decomposition of the vernix, during its passage to and deposit in the rectum. The fœtus swallows, from time to time, some of the liquor amnii, having the vernix swimming in it, and the hairs and horny scales pass unchanged along the intestinal tract. Whether any of the sebaceous matter is taken up by the lacteals, may, perhaps, be determined by microscopical examination of the intestinal villi of the fœtus; and it would be interesting to determine, by numerous examinations of the intestinal canal, at what period this swallowing of the liquor amnii commences. As the elements of the vernix are only suspended in the liquor in small quantities, a large quantity of this must be gradually swallowed to lead to the amount of meconium usually present. The water must be soon absorbed from the stomach, as it is never found in it. The greater portion is probably excreted by the kidneys, and again reaches the amnios. That it in nowise contributes material to the nourishment of the fœtus has been shown by Bischoff; but that does not prevent it serving some purpose in the economy. A regular examination of the entire contents of the intestinal canal, in numerous fœtuses of different ages, is required to elucidate these points; and especially would such examination be of interest in the case of monsters. That the acephalæ have no meconium has long been known, and has usually been attributed to the absence of the liver. This would, however, only explain the absence of its dark color; and the meconium will only be wanting when, by reason of the malformation of the intestinal canal, the reception and transport of the liquor amnii holding the vernix caseosa are prevented.—(*Wien Wochenschrift*, 1858, No. 32; from *Medical Times and Gazette*, No. 467.)

IV.—*Physiology of the Thymus Gland in a state of Health and of Disease.* By ALEX. FRIEDLEBEN, Frankfort, 1858, p. 366.

This is the title of an elaborate work on the thymus gland, founded upon a vast number of original observations. The most important conclusions are as follows:—

The thymus is a ductless gland, an aggregation of follicles, but moderately supplied with blood. Its nerves are chiefly those which belong to its vessels. It secretes a clear intercellular fluid of a serous character and containing numerous rounded nuclei and some cells. The nuclei pass unchanged into the general venous circulation. The follicles of the thymus are constantly being built up and destroyed.

The thymus increases in size from the time of its first appearance up to puberty, but after birth this growth is less and less marked, from year to year, while at the same time its secretory activity diminishes. The thymus disappears in adult life, undergoing oleaginous degeneration. This is first observed in the nerves. Then the arteries become narrowed and the veins dilated, so as to retard the circulation, and to produce finally the conversion of the whole organ into fatty matter. The thymus is sometimes absent in children otherwise healthy and well nourished.

The chemical elements of the thymus are water, albumen, gluten, sugar, lactic acid, pigment, fat, saline matter, and perhaps a trace of hypoxanthine. Of these, albumen, sugar, and salts, predominate in early life.

A large number of facts, observed by others or created by himself, finally enable the author to conclude that, during growth, the thymus gland aids in nutrition, in the preparation of blood, and in the general formation of tissues. M. Friedleben also furnishes the first analyses of this gland which have yet been made.

V.—*Increase of Muscle during Growth.* By J. BUDGE.

It has long been a matter of some interest to ascertain whether, during growth, and the hypertrophy caused by exercise, the muscular fibres of animal life increase in number or merely in size. M. Budge, in an interesting communication to the French Academy, states that the actual number of the fibres increases with every year during the period of growth, so that the increase of muscular tissue does not depend alone upon enlargement of the individual fibres of the muscles. M. Budge has also ascertained that starvation diminishes the number of the fibres, and notably lessens their size.—(*Journal de Physiologie ;* from *Comptes Rendus de l'Académie,* October, 1858.)

